# Initiation and/or re-initiation of drug use among people who use drugs in Vancouver, Canada from 2021 to 2022: a prospective cohort study

**DOI:** 10.1186/s13011-024-00624-8

**Published:** 2024-09-10

**Authors:** Anjali Sergeant, Paxton Bach, Jingxin Lei, Kora DeBeck, M-J Milloy, Kanna Hayashi

**Affiliations:** 1 Department of Medicine, University of British Columbia, 2775 Laurel Street, Vancouver, BC V5Z 1M9 Canada; 2 British Columbia Centre on Substance Use (BCCSU), 400-1045 Howe Street, Vancouver, BC V6Z 2A9 Canada; 3 School of Public Policy, Simon Fraser University, 515 West Hastings Street, Vancouver, BC V6B 5K3 Canada; 4 Faculty of Health Sciences, Simon Fraser University, 8888 University Drive, Burnaby, BC V5A 1S6 Canada

**Keywords:** COVID-19, Drug overdose, Substance-related disorders, Vulnerable populations

## Abstract

**Objectives:**

Widespread health service disruptions resulting from the COVID-19 pandemic coincided with a dramatic increase in overdose deaths among people who use drugs (PWUD) in Vancouver, Canada. Those with a history of injection drug use are known to be at heightened risk of substance-associated harms. Drug use patterns and associated sociodemographic and health care utilization trends have been understudied in this population since the pandemic onset. We sought to understand patterns of drug use initiation and/or re-initiation among people with a history of injection drug use (IVDU).

**Methods:**

Data were obtained from three harmonized prospective cohort studies of PWUD in Vancouver. Participants with a lifetime history of IVDU who responded to a survey between June 2021 and May 2022 were included. The primary outcome variable was a composite of substance use initiation and re-initiation over the study period, labelled as drug (re)-initiation. A multivariable generalized linear mixed-effects model was used to examine factors associated with self-reported (re)-initiation of substance use over the past six months.

**Results:**

Among 1061 participants, the median age was 47 years at baseline and 589 (55.5%) identified as men. In total, 183 (17.2%) participants reported initiating and/or re-initiating a drug, with 44 (4.1%) reporting new drug initiation and 148 (14.0%) reporting drug re-initiation (9 participants responded ‘yes’ to both). Overall, unregulated stimulants (e.g., crystal methamphetamine and cocaine) were the most common drug class (re-)initiated (n = 101; 55.2%), followed by opioids (n = 74; 40.4%) and psychedelics (n = 36; 19.7%). In the multivariable analysis, (re-)initiation of drug use was independently associated with recent IVDU (adjusted odds ratio [AOR] 2.62, 95% confidence interval [CI] 1.02, 6.76), incarceration (AOR 3.36, CI 1.12, 10.14) and inability to access addiction treatment (AOR 4.91, 95% CI 1.22, 19.75).

**Conclusions:**

In an era impacted by the intersecting effects of the COVID-19 pandemic and the overdose crisis, nearly one in five PWUD with a history of IVDU began using a new drug and/or re-started use of a previous drug. Those who reported drug (re-)initiation exhibited riskier substance use behaviours and reported difficulty accessing treatment services. Our findings underscore the need to provide additional resources to support this high-risk population.

**Supplementary Information:**

The online version contains supplementary material available at 10.1186/s13011-024-00624-8.

## Background

In addition to the substantial burden of morbidity and mortality, the COVID-19 pandemic heightened economic and social inequities, and reduced access to healthcare services across the globe [1]. In the North American context, the pandemic disproportionately impacted people who use drugs (PWUD), who faced the effects of a drug supply contaminated with highly potent synthetic opioids [2–5]. In British Columbia (BC), Canada, drug overdose deaths doubled in the years following the pandemic onset as access to health services was curtailed and the illicit drug market grew increasingly toxic [5, 6]. People who inject drugs face increased risks of harm associated with illicit or non-prescription substance use, including the transmission of viral infectious diseases, the development of systemic disease, and substance-related death [7]. The extent to which drug use patterns shifted among this high-risk subset of PWUD during this era is unclear. We sought to examine patterns of drug use initiation and/or re-initiation and associated sociodemographic characteristics, drug use behaviours, and service utilization trends among PWUD with a history of injection drug use (IVDU) from 2021 to 2022. We aim to offer insights into the ways in which pandemic-related restrictions may have shaped substance use patterns in Vancouver. Developing an understanding of substance use patterns during this period will enable our systems and policymakers to better support PWUD in future crises and service disruptions.

## Materials and methods

This study evaluates baseline and follow-up survey data from three harmonized prospective cohort studies of PWUD in Vancouver. The included cohort studies of community-recruited PWUD are the AIDS Care Cohort to Evaluate exposure to Survival Services (ACCESS), the Vancouver Injection Drug Users Study (VIDUS), and the At Risk Youth Study (ARYS). The ACCESS and VIDUS cohorts include individuals living within Greater Vancouver aged ≥ 18 years who reported unregulated drug use within the past month at study enrolment. The ACCESS cohort includes only those who are HIV positive at the time of enrollment. The VIDUS cohort is restricted to HIV-negative participants who reported IVDU at the time of enrollment, though participants may stop injecting drugs by the time of study follow-up [8]. The ARYS cohort includes participants aged 14–26 who report unregulated drug use in the past month and experience housing insecurity or access street-based services. Despite the differences in enrollment criteria, all cohorts include participants with a lifetime history of injection drug use in the Vancouver area. This term refers to participants who reported any current or previous engagement with drug injection at the first study visit during the study period.

As described previously [9, 10], participants were recruited by word-of-mouth or street outreach and provided informed consent to participate in the cohorts. They completed a baseline interviewer-administered questionnaire that documents sociodemographic characteristics, substance use patterns, and health care service utilization, which is repeated every six months after study enrollment. All cohorts have been reviewed and approved by the University of British Columbia/Providence Health Care research ethics board.

This study included participant data that was collected between June 2021 and May 2022. Due to the COVID-19 pandemic, interviews were held over the phone until March 2022 when in-person interviews were resumed. Participants with a lifetime history of IVDU who responded to the following survey question were included: “*In the last six months*,* have you initiated using a new drug or re-started using a drug that you had stopped using for at least six months*?” The primary outcome was defined as the initiation and/or re-initiation of drug use in the past six months (yes vs. no), which is labeled as drug (re)-initiation.

The definition of drug use included substances obtained through the illicit drug market as well as the non-medical use of prescribed pharmaceutical drugs. Multiple classes of substances were included within this composite outcome, including opioids (e.g. fentanyl, heroin, non-medical prescription use of opioids such as hydromorphone), stimulants (e.g. crystal methamphetamine, powder/crack cocaine), psychedelics (e.g. ecstasy, mushrooms), benzodiazepines (e.g. non-medical prescription use of lorazepam or diazepam) and other drugs that did not fall under any of the above classes. When completing the study interview, participants were given the option to report use of ‘other’ drugs, and those who responded affirmatively were asked to list the specific name of the drug(s). Substances that belonged to another drug category included in the survey were re-assigned prior to data analysis. A broad range of unregulated substances were included given the growing and near-ubiquitous practice of polysubstance use among high-risk subsets of PWUD, which increases the risk of drug-associated harms [11, 12]. Substances that are legal to consume in Canada, including alcohol and cannabis, were excluded from the primary outcome at the data analysis stage.

The following explanatory variables purported to impact drug (re-)initiation were included: age (continuous), gender (men vs. women or non-binary), ethnicity (white vs. Indigenous or persons of colour), residence in the Downtown Eastside, housing status, incarceration, IVDU, HIV status, non-fatal overdose, daily substance use (unregulated opioids, unregulated stimulants, cannabis and non-medical use of prescription opioids), drug or alcohol treatment, inability to access addictions treatment, and inability to access to health or social services. The Downtown Eastside is an urban area with high levels of marginalization and substance use [13]. Inability to access addictions treatment was determined by participants answering “yes” to the following question: “*In the past 6 months*,* have you tried to access any treatment program but were unable?”*. Inability to access health or social services was defined as participants responding “yes” to the question: “*In the last six months*,* was there a time you were in need of a service but could not obtain it?*”. The above definitions for the health and social service variables have been described and examined in the existing literature [7, 14]. All behavioural variables referred to the past six-month period at the time of interview. The six-month timeframe for drug (re-)initiation and explanatory variables reflects the biannual frequency of participant follow-up interviews, and this timeframe has been used in prior harmonized cohort analyses of this population [15, 16]. 

In order to analyze the data, we fit a multivariable generalized linear mixed-effects model to identify factors associated with drug (re-)initiation. All explanatory variables were included in the multivariable model and all p-values were two-sided. Sub-analyses were conducted to separately evaluate the initiation of a new drug (vs. no drug initiation) and the re-initiation of a drug (vs. no drug re-initiation), using the same multivariable modeling procedures as in the primary analyses. All statistical analyses were conducted in SAS 9.4.

## Results

Among 1061 eligible participants, the median age was 47 (1st and 3rd quartile: 36, 57) years, and 589 (55.5%) identified as men (Table 1). In terms of daily reported drug use, 376 (35.5%) participants used unregulated opioids, 18 (1.7%) used non-medical prescription opioids, 295 (27.9%) used stimulants, and 241 (22.7%) smoked or ingested cannabis products.

**Table 1:** Drug use (re-)initiation and associated participant characteristics

Participant Characteristics	Characteristics (n, %)		Bivariable Model		Multivariate Model	
	Overall	Yes to drug (re-)initiated	OR (95% CI)	p value	AOR (95% CI)	p value
	1061	183 (17.2)	--	--	--	--
Age (median (IQR))**	47 (36, 57)	43 (31, 54)	0.97 (0.95, 0.98)	< 0.001	0.98 (0.95, 1.00)	0.05
Men**^a^	589 (55.5)	87 (47.5)	0.65 (0.41, 1.02)	0.06	0.68 (0.39, 1.21)	0.19
White ethnicity*^b^	607 (57.6)	106 (58.2)	1.05 (0.73, 1.50)	0.81	1.14 (0.71–1.81)	0.59
Lack of housing**	158 (14.9)	30 (16.4)	1.45 (0.55, 3.77)	0.45	0.87 (0.26–2.92)	0.82
Reside in DTES**	524 (49.4)	80 (43.7)	1.32 (0.67, 2.60)	0.43	1.27 (0.55–2.95)	0.57
Incarceration**	51 (4.8)	10 (5.6)	4.30 (1.69, 10.97)	0.002	3.36 (1.12, 10.14)	0.03
Injection drug use**	536 (50.5)	114 (62.3)	3.62 (1.73, 7.55)	< 0.001	2.62 (1.02, 6.76)	0.05
HIV seropositive*	328 (30.9)	48 (26.2)	0.75 (0.51, 1.12)	0.16	1.09 (0.65–1.83)	0.75
Non-fatal overdose**	188 (17.8)	56 (30.8)	3.07 (1.32, 7.16)	0.009	2.01 (0.78, 5.20)	0.15
At least daily use of unregulated opioids**	376 (35.5)	66 (36.1)	1.78 (0.80, 3.96)	0.16	1.08 (0.39-3.00)	0.89
At least daily non-medical use of prescribed opioids**	18 (1.7)	6 (3.3)	5.15 (0.76, 35.05)	0.094	2.15 (0.21, 22.28)	0.52
At least daily use of stimulants**	295 (27.9)	65 (35.7)	1.30 (0.55, 3.06)	0.55	1.06 (0.41–2.77)	0.90
At least daily use of cannabis**	241 (22.7)	50 (27.3)	0.81 (0.33, 1.98)	0.64	1.12 (0.42–3.01)	0.82
Recent addictions treatment**	704 (66.7)	134 (73.6)	2.16 (0.89, 5.22)	0.09	1.64 (0.65, 4.11)	0.30
Inability to access addictions treatment**	59 (5.6)	17 (9.3)	5.35 (1.74, 16.49)	0.004	4.91 (1.22, 19.75)	0.03
Inability to access health or social services**	223 (21.2)	58 (32.0)	0.78 (0.26, 2.34)	0.66	0.55 (0.16–1.85)	0.33

OR: Odds Ratio. CI: Confidence Interval. AOR: Adjusted Odds Ratio. IQR: Interquartile range. DTES: Downtown Eastside. HIV: Human Immunodeficiency Virus

*Notes*

* Single asterisk refers to variables collected at the baseline study visit

** Double asterisk refers to variables collected at follow-up and refer to the past 6 month period

^a^Gender variable defined as man vs. woman or other

^b^ Ethnicity variable defined as white versus non-white

In total, 183 (17.2%) participants reported initiating or re-initiating a drug within the study period, with 44 (4.1%) reporting new drug initiation, 148 (14.0%) reporting drug re-initiation, and 9 participants reporting both.

Overall, stimulants were the most common drug type (re-)initiated (*n* = 101; 55.2%), followed by opioids (*n* = 74; 40.4%) and psychedelics (*n* = 36; 19.7%) (Table 2). Similarly, in the subgroup who declared drug re-initiation, the most common class of drug was stimulants (*n* = 91; 61.5%), followed by opioids (*n* = 60; 40.5%) and psychedelics (*n* = 19; 12.8%). For the smaller subgroup who reported new drug initiation, psychedelics were the most common drug type (*n* = 17; 38.6%) followed by opioids (*n* = 14; 31.8%) and then stimulants (*n* = 10; 22.7%).

**Table 2:** Classes of drugs (re-)initiated

Drug class*	Drug use initiation and/or re-initiation** N (%)	Initiation of new drug** N (%)	Re-initiation of prior drug** N (%)
	total n = 183	total n = 44	n = 148
Opioids	74 (40.4)	14 (31.8)	60 (40.5)
Stimulants	101 (55.2)	10 (22.7)	91 (61.5)
Benzodiazepines	4 (2.2)	4 (9.0)	0 (0)
Psychedelics	36 (19.7)	17 (38.6)	19 (12.8)
Other	14 (7.7)	7 (15.9)	7 (4.7)

*participants could select more than one drug type (re)-initiated

**drug use initiation and re-initiation defined as over the past 6-month period

As shown in the multivariable analysis (Table 1), initiation and/or re-initiation of drug use was independently associated with IVDU in the past six months (adjusted odds ratio [AOR] 2.62, 95% confidence interval [CI] 1.02, 6.76), incarceration status (AOR 3.36, 95% CI 1.12, 10.14) and the reported inability to access addictions treatment over the same timeframe (AOR 4.91, 95% CI 1.22, 19.75).

In the supplemental multivariable sub-analysis (Supplement Table 1), initiation of a new drug was independently associated with HIV serum positivity (AOR 5.18, 95% CI 1.36, 19.77), non-medical use of daily prescription opioids (AOR 10.97, 95% CI 1.18, 101.75), and inability to access addictions treatment (AOR 13.85, 95% CI 1.33, 144.10). Multivariable analysis focused on re-initiation of a previously used drug did not reveal any independent associations (Supplement Table 2).

## Discussion

In this longitudinal study of individuals with a lifetime history of IVDU in Vancouver, nearly one in five participants responded ‘yes’ to initiating or re-initiating drug use between 2021 and 2022. Over 80% of these participants re-initiating a drug that had been used previously. Our findings demonstrate that a significant proportion of participants (re)-initiated substance use in an era characterized by the intersecting crises of the COVID-19 pandemic and fatal opioid-related overdoses.

Stimulants were the most commonly (re-)initiated drug class in our overall cohort, particularly among those who endorsed re-initiating use of a previous drug. This finding aligns with evidence that stimulant use among PWUD is rising in Vancouver and North America more broadly, often co-ingested with opioids [17, 18]. PWUD who face housing insecurity may turn to stimulants as a means to stay alert and “protect themselves and their belongings,” while others may engage in stimulant use to temper the sedating effects of opioids [19]. The rise in stimulant use is especially concerning given the paucity of evidence-based treatments for stimulant dependence [20]. Interestingly, among the subgroup of participants who started using a drug type for their first time, psychedelics were the most commonly reported class. With the advent of decriminalization policies and increased accessibility to psychedelics (i.e. magic mushroom dispensaries), psychedelic use among people who use drugs warrants further research [21]. 

Individuals in our cohort who reported drug (re-)initiation were more likely to report IVDU, recent incarceration, and experienced greater difficulty accessing addiction treatment within the last six months. Injection drug use, previous incarceration, and lack of access to addiction treatment are known predictors of fatal overdoses among PWUD [22–24]. In Vancouver, a dramatic rise in overdose-related deaths has occurred since 2020, the onset of the COVID-19 pandemic [4, 5]. Several pandemic-related factors have been cited as likely contributors to these deaths, including drug market disruptions that created a more toxic street supply, interruptions in health and social service provision, and widespread social isolation [3, 25]. 

There is an unmet need for addiction treatment services in Canada, an issue that preceded COVID-19 but was exacerbated during this time [26, 27]. Opioid agonist therapy (OAT), residential treatment services, and counselling from addictions-trained healthcare professionals became more challenging for some to access during the pandemic [26, 28]. While approximately two-thirds of our overall study cohort reported that they had received treatment for substance use disorder within the last six months, over 5% reported that they had tried unsuccessfully to seek addictions treatment. The strong network of treatment services and drug user advocacy groups in Vancouver’s Downtown Eastside neighbourhood that has developed over decades likely mitigated more extensive gaps in care [28, 29]. In other regions of the country, a higher proportion of PWUD may have faced treatment barriers for substance use disorders during this period [28]. 

Our findings suggest that reduced access to addiction treatment services among PWUD in Vancouver may have contributed to higher-risk drug (re)-initiations following the onset of the COVID-19 pandemic. Prior evidence demonstrates that PWUD who experience the most difficulty accessing treatment services often face structural barriers such as housing instability [30]. Local provider responses to the pandemic, such as lengthened and take-home OAT prescriptions and expanded telehealth services, likely helped to expand treatment accessibility to underserved populations with substance use disorders [31, 32]. Despite these important efforts, our study supports evidence that the reduced accessibility of treatment services during the pandemic may have contributed to an increase in drug-associated harms and deaths among the most vulnerable [33]. 

In our sub-analysis focused solely on the initiation of a new drug, those who reported novel use of a drug were more likely to carry HIV seropositivity. It is established that PWUD living with HIV face higher morbidity and mortality rates compared with their peers [34]. Following the onset of the COVID-19 pandemic, a Vancouver-based study found that treatment gaps among HIV-positive individuals widened, and viremia levels increased among those with no fixed address [35]. For some, pandemic-related scarcities affected the delivery of high-quality HIV care. People using drugs and living with HIV faced multiple challenges related to health service access during the pandemic, stressors which may have contributed to the riskier use of new substances. Our study highlights the need to prioritize the care of PWUD with HIV amidst healthcare crises.

The study has several limitations. Given its observational nature, there is a potential for unmeasured confounders and we are unable to draw causal relationships or confirm the directionality of the associations between drug use (re-)initiation and participant characteristics. The study period covered a limited timeframe with respect to COVID-19 and is unable to ascertain to what degree the pandemic impacted the drug use patterns described. By evaluating drug use initiation and re-initiation as a composite outcome, the study risks over-interpreting the associations between drug patterns and the explanatory variables included. The generalizability of our findings may be limited as the study focused on participants in Vancouver, a city with a high density of unhoused people using drugs and an established network of outreach services, which may impact experiences of drug use. The study was restricted to people with a lifetime history of IVDU, which narrows the scope to a particularly high-risk subset of PWUD and contributes to homogeneity in the analyses.

## Conclusions

We found that a substantial proportion of people with a lifetime history of IVDU (re-)initiated drug use from 2021 to 2022, a period impacted by the intersecting COVID-19 pandemic and overdose crisis. Those who started using a new drug or re-started using a drug were more likely to report risky drug use behaviours and experience difficulty accessing addictions treatment. A more nuanced understanding of the social, behavioural, and structural factors that may have contributed to drug use (re-)initiation among this highly vulnerable population will be important in identifying areas for targeted intervention.

## Electronic supplementary material

Below is the link to the electronic supplementary material.

Supplementary Material 1

Supplementary Material 2

## Data Availability

The datasets generated and/or analysed during the current study are not publicly available as they contain sensitive information about structurally marginalized and vulnerable individuals and populations. Datasets are available from the corresponding author on reasonable request, and with the permission of the University of British Columbia/Providence Health Care Research Ethics Board.

## Abbreviations

PWUD: People who use drugs
BC: British Columbia
IVDU: Injection drug use
VIDUS: Vancouver Injection Drug Users Study
ARYS: At Risk Youth Study
ACCESS: AIDS Care Cohort to Evaluate exposure to Survival Services

## References

[1] Barron GC, Laryea-Adjei G, Vike-Freiberga V, et al. Safeguarding people living in vulnerable conditions in the COVID-19 era through universal health coverage and social protection. Lancet Public Health Jan. 2022;7(1):e86–92. 10.1016/S2468-2667(21)00235-8. 10.1016/S2468-2667(21)00235-8 PMC8665842 34906331

[2] Murphy SM, Yoder J, Pathak J, Avery J. Healthcare utilization patterns among persons who use drugs during the COVID-19 pandemic. J Subst Abuse Treat Feb. 2021;121:108177. 10.1016/j.jsat.2020.108177. 10.1016/j.jsat.2020.108177 PMC7575533 33109432

[3] Jacka BP, Janssen T, Garner BR, et al. Impacts of the COVID-19 pandemic on healthcare access among patients receiving medication for opioid use disorder. Drug Alcohol Depend Apr. 2021;01:221:108617. 10.1016/j.drugalcdep.2021.108617. 10.1016/j.drugalcdep.2021.108617 PMC7883735 33647590

[4] Striley CW, Hoeflich CC. Converging public health crises: substance use during the coronavirus disease 2019 pandemic. Curr Opin Psychiatry Jul. 2021;01(4):325–31. 10.1097/YCO.0000000000000722. 10.1097/YCO.0000000000000722 PMC8183237 34001699

[5] Palis H, Bélair MA, Hu K, Tu A, Buxton J, Slaunwhite A. Overdose deaths and the COVID - 19 pandemic in British Columbia, Canada. Drug Alcohol Rev. 2022;41(4):912–7. 34908203 10.1111/dar.13424

[6] Illicit Drug Toxicity Deaths in BC: January 1. 2012 – December 31, 2022. Ministry of Public Safety & Solicitor General. Accessed May 5, 2023. https://www2.gov.bc.ca/assets/gov/birth-adoption-death-marriage-and-divorce/deaths/coroners-service/statistical/illicit-drug.pdf

[7] Prangnell A, Daly-Grafstein B, Dong H, et al. Factors associated with inability to access addiction treatment among people who inject drugs in Vancouver, Canada. Subst Abuse Treat Prev Policy. 2016;11(1):1–8. 26916425 10.1186/s13011-016-0053-6 PMC4766680

[8] Dong H, Hayashi K, Singer J, et al. Trajectories of injection drug use among people who use drugs in Vancouver, Canada, 1996–2017: growth mixture modeling using data from prospective cohort studies. Addict Dec. 2019;114(12):2173–86. 10.1111/add.14756. 10.1111/add.14756 PMC7498269 31328354

[9] Strathdee SA, Patrick DM, Currie SL, et al. Needle exchange is not enough: lessons from the Vancouver injecting drug use study. AIDS. 1997;11(8):59–65. 9223727 10.1097/00002030-199708000-00001

[10] Wood E, Stoltz J-A, Montaner JS, Kerr T. Evaluating methamphetamine use and risks of injection initiation among street youth: the ARYS study. Harm Reduct J. 2006;3(18). 10.1186/1477-7517-3-18 PMC1481558 16723029

[11] Cicero TJ, Ellis MS, Kasper ZA. Polysubstance use: a broader understanding of substance use during the opioid crisis. Am J Public Health. 2020;110(2):244–50. 31855487 10.2105/AJPH.2019.305412 PMC6951387

[12] Karamouzian M, Cui Z, Hayashi K, et al. Longitudinal latent polysubstance use patterns among a cohort of people who use opioids in Vancouver, Canada. Drug Alcohol Rev Sep. 2023;42(6):1493–503. 10.1111/dar.13690. 10.1111/dar.13690 PMC10705814 37282794

[13] Linden IA, Mar M, Werker GR, Jang K, Krausz M. Research on a vulnerable neighborhood—the Vancouver Downtown Eastside from 2001 to 2011. J Urb Health. 2013;90:559–73. 10.1007/s11524-012-9771-x PMC3665976 23161093

[14] Wang L, Panagiotoglou D, Min JE, et al. Inability to access health and social services associated with mental health among people who inject drugs in a Canadian setting. Drug Alcohol Depend Nov. 2016;01:168:22–9. 10.1016/j.drugalcdep.2016.08.631. 10.1016/j.drugalcdep.2016.08.631 PMC5086265 27610937

[15] Cui Z, Hayashi K, Bach P, Dong H, Milloy MJ, Kerr T. Predictors of crystal methamphetamine use initiation or re-initiation among people receiving opioid agonist therapy: a prospective cohort study. Drug Alcohol Depend Nov. 2022;01:240:109624. 10.1016/j.drugalcdep.2022.109624. 10.1016/j.drugalcdep.2022.109624 PMC9886065 36116155

[16] Damon W, McNeil R, Milloy MJ, Nosova E, Kerr T, Hayashi K. Residential eviction predicts initiation of or relapse into crystal methamphetamine use among people who inject drugs: a prospective cohort study. J Public Health (Oxf) Mar. 2019;01(1):36–45. 10.1093/pubmed/fdx187. 10.1093/pubmed/fdx187 PMC6490768 29425315

[17] Hayashi K, Milloy M-J, Lysyshyn M, et al. Substance use patterns associated with recent exposure to fentanyl among people who inject drugs in Vancouver, Canada: a cross-sectional urine toxicology screening study. Drug Alcohol Depend. 2017;183:1–6. 29220642 10.1016/j.drugalcdep.2017.10.020 PMC5803313

[18] Ciccarone D. The rise of illicit fentanyls, stimulants and the fourth wave of the opioid overdose crisis. Curr Opin Psychiatry Jul. 2021;01(4):344–50. 10.1097/YCO.0000000000000717. 10.1097/YCO.0000000000000717 PMC8154745 33965972

[19] Canêdo J, Sedgemore KO, Ebbert K, et al. Harm reduction calls to action from young people who use drugs on the streets of Vancouver and Lisbon. Harm Reduct J May. 2022;04(1):43. 10.1186/s12954-022-00607-7. 10.1186/s12954-022-00607-7 PMC9064719 35505320

[20] Ronsley C, Nolan S, Knight R et al. Treatment of stimulant use disorder: a systematic review of reviews. PLoS ONE. 2020;15(6). 10.1371/journal.pone.0234809 PMC7302911 32555667

[21] Zannese K. High time? Psychedelics on cannabis-like fast track to legalization. CMAJ Dec. 2022;19(49):E1695–6. 10.1503/cmaj.1096029. 10.1503/cmaj.1096029 PMC9829048 36535680

[22] Lyons R, Yule A, Schiff D, Bagley S, Wilens T. Risk factors for drug overdose in young people: a systematic review of the literature. J Child Adolesc Psychopharmacol. 2019;29(7):487–97. 31246496 10.1089/cap.2019.0013 PMC6727478

[23] Cano M, Oh S, Osborn P, et al. County-level predictors of US drug overdose mortality: a systematic review. Drug Alcohol Depend Jan. 2023;01:242:109714. 10.1016/j.drugalcdep.2022.109714. 10.1016/j.drugalcdep.2022.109714 36463764

[24] Gan WQ, Kinner SA, Nicholls TL, et al. Risk of overdose-related death for people with a history of incarceration. Addict Jun. 2021;116(6):1460–71. 10.1111/add.15293. 10.1111/add.15293 33047844

[25] Nguyen T, Buxton JA. Pathways between COVID-19 public health responses and increasing overdose risks: a rapid review and conceptual framework. Int J Drug Policy Jul. 2021;93:103236. 10.1016/j.drugpo.2021.103236. 10.1016/j.drugpo.2021.103236 PMC8545050 33838990

[26] Russell C, Ali F, Nafeh F, Rehm J, LeBlanc S, Elton-Marshall T. Identifying the impacts of the COVID-19 pandemic on service access for people who use drugs (PWUD): a national qualitative study. J Subst Abuse Treat. 2021;129. 10.1016/j.jsat.2021.108374 PMC9757985 34080545

[27] Fischer B, Murphy Y, Rudzinski K, MacPherson D. Illicit drug use and harms, and related interventions and policy in Canada: a narrative review of select key indicators and developments since 2000. Int J Drug Policy. 2016;27:23–35. 26359046 10.1016/j.drugpo.2015.08.007

[28] Mitra S, Bouck Z, Larney S, et al. The impact of the COVID-19 pandemic on people who use drugs in three Canadian cities: a cross-sectional analysis. Harm Reduct J May. 2024;16(1):94. 10.1186/s12954-024-00996-x. 10.1186/s12954-024-00996-x PMC11097551 38750575

[29] Wood E, Kerr T. What do you do when you hit rock bottom? Responding to drugs in the city of Vancouver. Int J Drug Policy. 2006;17(2):55–60. 10.1016/j.drugpo.2005.12.007

[30] Hyshka E, Anderson JT, Wild TC. Perceived unmet need and barriers to care amongst street-involved people who use illicit drugs. Drug Alcohol Rev May. 2017;36(3):295–304. 10.1111/dar.12427. 10.1111/dar.12427 27242102

[31] Krawczyk N, Fawole A, Yang J, Tofighi B. Early innovations in opioid use disorder treatment and harm reduction during the COVID-19 pandemic: a scoping review. Addict Sci Clin Pract Nov. 2021;13(1):68. 10.1186/s13722-021-00275-1. 10.1186/s13722-021-00275-1 PMC8590133 34774106

[32] MacKinnon L, Socías ME, Bardwell G. COVID-19 and overdose prevention: challenges and opportunities for clinical practice in housing settings. J Subst Abuse Treat Dec. 2020;119:108153. 10.1016/j.jsat.2020.108153. 10.1016/j.jsat.2020.108153 PMC7532988 33032862

[33] Ali F, Russell C, Nafeh F, Rehm J, LeBlanc S, Elton-Marshall T. Changes in substance supply and use characteristics among people who use drugs (PWUD) during the COVID-19 global pandemic: a national qualitative assessment in Canada. Int J Drug Policy Jul. 2021;93:103237. 10.1016/j.drugpo.2021.103237. 10.1016/j.drugpo.2021.103237 PMC9759688 33893026

[34] Altice FL, Kamarulzaman A, Soriano VV, Schechter M, Friedland GH. Treatment of medical, psychiatric, and substance-use comorbidities in people infected with HIV who use drugs. Lancet Jul. 2010;31(9738):367–87. 10.1016/S0140-6736(10)60829-X. 10.1016/S0140-6736(10)60829-X PMC4855280 20650518

[35] Munasinghe LL, Yin W, Nathani H, et al. The impact of the COVID-19 pandemic on HIV treatment gap lengths and viremia among people living with HIV British Columbia, Canada, during the COVID-19 pandemic: are we ready for the next pandemic? Soc Sci Med Jun. 2024;350:116920. 10.1016/j.socscimed.2024.116920. 10.1016/j.socscimed.2024.116920 38703468

