# Supplementary material for: Initiation and/or re-initiation of drug use among people who use drugs in Vancouver, Canada from 2021 to 2022: a prospective cohort study
Source: Subst Abuse Treat Prev Policy. 2024 Sep 10;19:42. doi: 10.1186/s13011-024-00624-8 (PMC11385492; doi:10.1186/s13011-024-00624-8)
Supplement: Supplementary file 1 — Supplementary Material 1 [file 13011_2024_624_MOESM1_ESM.docx]

**Supplement Table 1***:* Drug use initiation alone and associated participant characteristics

|  | Baseline Characteristics (n, %) | | Bivariable Model | | Multivariate Model | |
| --- | --- | --- | --- | --- | --- | --- |
| Participant Characteristics | Overall | Yes to drug initiated | OR (95% CI) | p value | AOR (95% CI) | p value |
|  | 1061 | 44 (4.2%) | -- | -- | -- | -- |
| Age (median (IQR))** | 47 (36, 57) | 45 (32, 53) | 0.98 (0.96, 1.00) | 0.03 | 0.97 (0.92, 1.03) | 0.29 |
| Men** ^a^ | 589 (55.5) | 20 (45.5) | 0.55 (0.14, 2.15) | 0.39 | 0.70 (0.15, 3.35) | 0.66 |
| White ethnicity*^b^ | 607 (57.6) | 24 (54.6) | 0.82 (0.45, 1.49) | 0.52 | 1.46 (0.50, 4.27) | 0.49 |
| Lack of housing** | 158 (14.9) | 6 (13.6) | 1.70 (0.20, 14.7) | 0.63 | 0.47 (0.03, 7.68) | 0.60 |
| Reside in DTES** | 524 (49.4) | 18 (40.9) | 1.92 (0.37, 10.0) | 0.44 | 2.23 (0.31, 15.91) | 0.42 |
| Incarceration** | 51 (4.8) | 3 (6.82) | 5.12 (1.32, 19.90) | 0.02 | 5.49 (0.70, 42.87) | 0.10 |
| Injection drug use** | 536 (50.5) | 24 (54.6) | 1.71 (0.30, 9.70) | 0.54 | 1.21 (0.15, 9.41) | 0.86 |
| HIV seropositive* | 328 (30.9) | 18 (40.9) | 1.69 (0.92, 3.10) | 0.09 | 5.18 (1.36,19.77) | 0.02 |
| Non-fatal overdose** | 188 (17.8) | 18 (41.9) | 3.48 (0.51, 23.80) | 0.20 | 3.78 (0.41, 34.70) | 0.24 |
| At least daily use of unregulated opioids** | 376 (35.5) | 15 (34.1) | 1.44 (0.27, 7.72) | 0.67 | 0.85 (0.08, 8.73) | 0.89 |
| At least daily non-medical use of prescribed opioids** | 18 (1.7) | 3 (6.8) | 16.76 (3.13, 89.76) | 0.001 | 10.97 (1.18, 101.75) | 0.04 |
| At least daily use of stimulants** | 295 (27.9) | 18 (41.9) | 2.09 (0.36, 11.95) | 0.41 | 1.28 (0.17, 9.55) | 0.81 |
| At least daily use of cannabis** | 241 (22.7) | 9 (20.5) | 1.28 (0.25, 6.67) | 0.77 | 1.34 (0.19, 9.66) | 0.77 |
| Recent addictions treatment** | 704 (66.7) | 28 (63.6) | 0.63 (0.16, 2.50) | 0.51 | 0.33 (0.05, 2.27) | 0.26 |
| Inability to access addictions treatment** | 59 (5.6) | 7 (15.9) | 16.25 (2.74-96.46) | 0.002 | 13.85 (1.33, 144.10) | 0.03 |
| Inability to access health or social services** | 223 (21.2) | 16 (36.4) | 3.08 (0.55, 17.32) | 0.20 | 2.07 (0.27, 16.23) | 0.49 |

| OR: Odds Ratio. CI: Confidence Interval. AOR: Adjusted Odds Ratio. IQR: Interquartile range. DTES: Downtown Eastside. HIV: Human Immunodeficiency Virus.  *Notes*  * Single asterisk refers to variables collected at the baseline study visit  ** Double asterisk refers to variables collected at follow-up and refer to the past 6 month period  ^a^ Gender variable defined as man vs. woman or other  ^b^ Ethnicity variable defined as white versus non-white |
| --- |
