# Supplementary material for: Initiation and/or re-initiation of drug use among people who use drugs in Vancouver, Canada from 2021 to 2022: a prospective cohort study
Source: Subst Abuse Treat Prev Policy. 2024 Sep 10;19:42. doi: 10.1186/s13011-024-00624-8 (PMC11385492; doi:10.1186/s13011-024-00624-8)
Supplement: Supplementary file 2 — Supplementary Material 2 [file 13011_2024_624_MOESM2_ESM.docx]

**Supplement Table 2***:* Drug use re-initiation alone and associated participant characteristics

|  | Baseline Characteristics (n, %) | | Bivariable Model | | Multivariate Model | |
| --- | --- | --- | --- | --- | --- | --- |
| Participant Characteristics | Overall | Yes to drug re-initiated | OR (95% CI) | p value | AOR (95% CI) | p value |
|  | 1061 | 148 (13.9%) | -- | -- | -- | -- |
| Age (median (IQR))** | 47 (36, 57) | 42 (31, 55) | 0.97 (0.95, 0.98) | <0.001 | 0.98 (0.95, 1.00) | 0.06 |
| Men**^a^ | 589 (55.5) | 69 (46.6) | 0.71 (0.44, 1.12) | 0.14 | 0.67 (0.39, 1.14) | 0.14 |
| White ethnicity*^b^ | 607 (57.6) | 87 (59.2) | 1.12 (0.77, 1.64) | 0.54 | 1.13 (0.72, 1.79) | 0.58 |
| Lack of housing** | 158 (14.9) | 25 (16.9) | 1.33 (0.45, 3.89) | 0.61 | 1.04 (0.28, 3.84) | 0.96 |
| Reside in DTES** | 524 (49.4) | 67 (45.3) | 1.20 (0.57, 2.54) | 0.63 | 1.22 (0.49, 3.03) | 0.67 |
| Incarceration** | 51 (4.8) | 7 (4.83) | 3.16 (1.18, 8.50) | 0.02 | 1.86 (0.60, 5.73) | 0.28 |
| Injection drug use** | 536 (50.5) | 94 (63.5) | 3.66 (1.53, 8.80) | 0.004 | 2.82 (0.92, 8.67) | 0.07 |
| HIV seropositive* | 328 (30.9) | 33 (22.3) | 0.57 (0.37, 0.88) | 0.01 | 0.80 (0.47, 1.36) | 0.41 |
| Non-fatal overdose** | 188 (17.8) | 40 (27.2) | 2.31 (0.91, 5.88) | 0.08 | 1.25 (0.44, 3.56) | 0.68 |
| At least daily use of unregulated opioids** | 376 (35.5) | 56 (37.8) | 1.84 (0.79, 4.27) | 0.16 | 1.28 (0.43, 3.81) | 0.65 |
| At least daily non-medical use of prescribed opioids** | 18 (1.7) | 3 (2.0) | 1.80 (0.13, 25.03) | 0.66 | 0.39 (0.00, 42.41) | 0.69 |
| At least daily use of stimulants** | 295 (27.9) | 50 (34.0) | 1.08 (0.41, 2.86) | 0.87 | 0.86 (0.29, 2.57) | 0.79 |
| At least daily use of cannabis** | 241 (22.7) | 44 (29.7) | 0.75 (0.27, 2.09) | 0.59 | 1.16 (0.40, 3.38) | 0.79 |
| Recent addictions treatment** | 704 (66.7) | 111 (75.5) | 2.91 (0.97, 8.72) | 0.06 | 2.44 (0.79, 7.53) | 0.12 |
| Inability to access addictions treatment** | 59 (5.6) | 12 (8.16) | 1.87 (0.42, 8.41) | 0.41 | 2.65 (0.57, 12.32) | 0.22 |
| Inability to access health or social services** | 223 (21.2) | 46 (31.5) | 0.48 (0.11, 2.02) | 0.32 | 0.40 (0.10, 1.68) | 0.21 |

| OR: Odds Ratio. CI: Confidence Interval. AOR: Adjusted Odds Ratio. IQR: Interquartile range. DTES: Downtown Eastside. HIV: Human Immunodeficiency Virus.  *Notes*  * Single asterisk refers to variables collected at the baseline study visit  ** Double asterisk refers to variables collected at follow-up and refer to the past 6 month period  ^a^Gender variable defined as man vs. woman or other  ^b^ Ethnicity variable defined as white versus non-white |
| --- |
